# Reactivation of dead sulfide species in lithium polysulfide flow battery for grid scale energy storage

**DOI:** 10.1038/s41467-017-00537-0

**Published:** 2017-09-06

**Authors:** Yang Jin, Guangmin Zhou, Feifei Shi, Denys Zhuo, Jie Zhao, Kai Liu, Yayuan Liu, Chenxi Zu, Wei Chen, Rufan Zhang, Xuanyi Huang, Yi Cui

**Affiliations:** 1 Department of Materials Science and Engineering, Stanford University, Stanford, CA 94305 USA; 2 School of Electrical Engineering, Zhengzhou University, Zhengzhou, 450001 China; 3 State Key Lab of Electrical Insulation and Power Equipment, School of Electrical Engineering, Xi’an Jiaotong University, Xi’an, 710049 China; 4 Stanford Institute for Materials and Energy Sciences, SLAC National Accelerator Laboratory, 2575 Sand Hill Road, Menlo Park, CA 94025 USA

## Abstract

Lithium polysulfide batteries possess several favorable attributes including low cost and high energy density for grid energy storage. However, the precipitation of insoluble and irreversible sulfide species on the surface of carbon and lithium (called “dead” sulfide species) leads to continuous capacity degradation in high mass loading cells, which represents a great challenge. To address this problem, herein we propose a strategy to reactivate dead sulfide species by reacting them with sulfur powder with stirring and heating (70 °C) to recover the cell capacity, and further demonstrate a flow battery system based on the reactivation approach. As a result, ultrahigh mass loading (0.125 g cm^–3^, 2 g sulfur in a single cell), high volumetric energy density (135 Wh L^–1^), good cycle life, and high single-cell capacity are achieved. The high volumetric energy density indicates its promising application for future grid energy storage.

## Abstract (web summary)

Lithium polysulfide batteries suffer from the precipitation of insoluble and irreversible sulfide species on the surface of carbon and lithium. Here the authors show a reactivation strategy by a reaction with cheap sulfur powder under stirring and heating to recover the cell capacity.

## Introduction

The urgent requirement to develop and integrate renewable energy such as wind and solar into the grid has driven the intense demand for high energy storage systems for grid scale energy storage^1–3^. Electrochemical energy storage, with the benefits of pollution-free operation, high round-trip efficiency, and flexible power, has been regarded as one of the most effective ways to solve the problem of intermittent renewable energy penetration^4^. Commercialized sodium–sulfur (Na–S) batteries have already been implemented for grid applications to regulate peak load and frequency, however, it faces a big safety challenge due to its high working temperature (300–350 °C)^5^. Its high cost (300$ kW h^−1^) also limits its large-scale application^6^. Vanadium redox flow batteries are promising but are still limited by their low energy density (< 50 Wh kg^−1^), relatively high cost and environmental toxicity^7–9^. Other systems such as liquid metal batteries are emerging, is promising but inevitably face the barrier of high working temperature^10^.

In order to satisfy future large-scale renewable energy storage applications, low cost (<100$ kW h^−1^), high energy density (>100 Wh kg^−1^), and safe (room temperature operation) electrochemical energy storage systems are urgently needed^6, 11^. Lithium–sulfur (Li–S) batteries, with a theoretical energy density of 2600 Wh kg^−1^, are one of the most promising candidates for next-generation rechargeable lithium batteries^12, 13^. However, the low electrical conductivity of sulfur/Li_2_S, deposition of non-soluble and insulating Li_2_S/Li_2_S_2_ on the electrodes, volume change during cycling, and self-discharge and shuttle effect make it difficult to move toward industrial application^14, 15^, especially for high mass loading sulfur electrodes. Many strategies have been proposed to control soluble lithium polysulfides including the design of various carbon–sulfur cathodes or other architectures to confine sulfur^16–20^, exploration of new electrolytes or additives^21, 22^, modification of battery configuration^23, 24^, and protection of the metallic lithium anode^25–27^. Although these strategies can achieve improved electrochemical performance, these issues have still not been entirely solved, and new directions for Li-S batteries are still in development^28–30^.

Our group previously reported on lithium polysulfide (LPS) semi-liquid battery^31^, in which liquid polysulfide was used as cathode and metallic lithium as anode, demonstrating high energy density and compatibility with flow battery design (Supplementary Table 1). With the addition of lithium nitrate (LiNO_3_), a passivation layer will form on the surface of metallic lithium, suppressing parasitic reactions between polysulfide and lithium. No ion-selective membrane was needed thus the cost was reduced. Most of the characterization of the electrochemical performance of LPS batteries were conducted using coin cells, resulting in low mass loading of sulfur, which is not representative of real-world conditions. In the coin cell configuration, it is also difficult to evaluate the full battery performance and not compatible with the semi-liquid flow battery concept.

To better realize the high capacity and high mass loading that are necessary for an industrial set-up, a new battery tank that is suitable for large scale and semi-liquid flow demonstration was designed. During our preliminary high mass loading performance test, we found that the cell capacity was not stable and decayed very quickly. After disassembling the battery tank, it was surprising to find large quantities of insoluble sulfur species deposited on both lithium and cathode (carbon felt) electrodes. These insoluble species result from the low solubility of low-order polysulfides like Li_2_S_2_/Li_2_S. As the metallic lithium used in the LPS battery is in excess compared with the quantity of sulfur, the consumption of metallic lithium reduces the S/Li ratio and causes precipitation of the insoluble low-order polysulfides. These insoluble sulfide species (Li_2_S_*x*_, mainly Li_2_S/Li_2_S_2_), called “dead” sulfide species, are inactive and cannot contribute capacity in the following cycles. The conversion of soluble high-order polysulfides into insoluble low-order polysulfides (dead sulfide species) is the main cause of fast capacity decay and finally loss of electrochemical activity.

To address the above challenges, herein we propose a method for the reactivation of dead sulfide species in the cell through stirring and heating them with sulfur at a relatively low temperature (70 °C). The main idea of reactivation is to use additional cheap sulfur powder to react with the dead sulfide species in order to recover the lost capacity. By virtue of our battery tank design, the reactivation process can be implemented without disassembling the battery. The single-cell capacity can reach as high as 0.9 Ah with a corresponding volumetric energy density of 95 Wh L^−1^ (3 M Li_2_S_8_), approximately four times higher than that of vanadium flow battery (25 Wh L^−1^). With high concentration Li_2_S_8_ (5 M), the volumetric energy density can reach as high as 135 Wh L^−1^. It is noted that the energy density and specific energy here are calculated based on the real cell volume and weight (including polysulfide catholyte, LiNO_3_ additive, lithium anode, carbon felt, and separator. The theoretical and real energy density calculation is presented in Supplementary Notes 1 and 2. Recipe is shown in Supplementary Table 2). Excellent performance over 110 cycles was attained with reactivation every 50 cycles, demonstrating an ultrahigh mass loading of 0.125 g cm^−3^ (2 g sulfur in single cell). To the best of our knowledge, this is the first time that such high mass loading with stable capacity is reported for LPS batteries, which is distinctly different from test results derived from coin cells. To further verify the possibility of its practical use in semi-liquid flow systems for future large-scale energy storage, a LPS flow battery system was successfully demonstrated employing our battery tank and reactivation strategy. A capacity of 1 Ah and long cycle life over 300 cycles are achieved with a reactivation tank connected with the battery system through a circulation pump, which gives a new prospect for low cost, high energy density, and stable grid scale energy storage.

## Results

### LPS battery configuration for reactivation

**Fig. 1:**
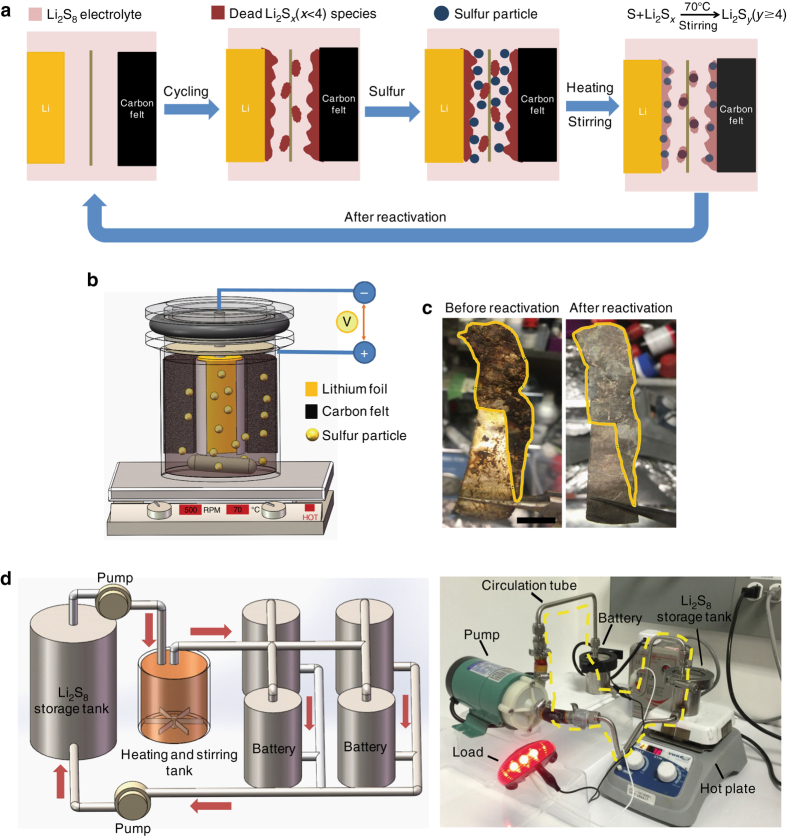
Schematic and optical image of reactivation of LPS battery using stirring and heating method. **a** Schematic of the reactivation process by reaction of sulfur particles with dead sulfide species under stirring and heating condition. **b** Design of LPS battery tank with reactivation function on a stirring and heating hot plate. (A magnetic stir bar was placed at the bottom of the battery tank for stirring and additional sulfur powder was placed on the bottom before sealing). **c** Optical image of lithium foil before and after reactivation (heating and stirring at 70 °C for 3 h). Dead sulfide species are shown on the surface of lithium foil marked by the outlined *yellow area* (before reactivation, after 50 cycles). After reactivation, almost all of the dead sulfide species disappeared. (*Scale bar*, 2 cm) **d** Schematic drawing of LPS flow battery system for future grid energy storage (left). and optical image of LPS flow battery system (with the function of reactivation) demonstration based on above schematic drawing (right)

To address the problem of dead sulfide species deposition on the lithium and carbon electrodes, reactivation via heating and stirring at a relatively low temperature (70 °C) was conducted to recycle the dead sulfide species by reacting them with sulfur powder in order to recover the cell capacity. We hypothesize that such activation is possible since it is similar to how we prepare polysulfide solution by using Li_2_S and sulfur powder mixed in ether solvent at elevated temperature^28, 31^. This approach has two obvious benefits—cheap sulfur powder can be used to increase the capacity while also removing the dead sulfide species from the surface of the electrodes. The process is illustrated in Fig. 1a. After prolonged cycling, dead Li_2_S_*x*_ deposits on both the surface of the lithium foil and the carbon felt. Then the cell was moved to a hot plate with heating and stirring functions (Fig. 1b), the stirring rate here was 500 RPM for stable and homogeneously stirring (this rate is variable according to the battery tank size and stirring bar size). The dead sulfide species react with sulfur during heating and stirring and convert to soluble high-order polysulfides, like Li_2_S_8_, Li_2_S_6_, and Li_2_S_4_, thus becoming reactivated and increasing the cell capacity. Based on our design, the sulfur added was sufficient for reactivation, and the dead sulfide species were mainly converted to Li_2_S_8_, while Li_2_S_6_ and Li_2_S_4_ also exist due to the equilibrium in the electrolyte. It was apparent that coin cell or pouch cell configurations used in previous research was not suitable for demonstrating the reactivation concept so to verify the above effect in LPS battery, a new battery configuration was therefore designed for high mass loading tests. As shown in Fig. 1b, a stainless steel tank was made for cell assembly (optical image of different sized tanks in Supplementary Fig. 1). The positive and negative sides were electrically insulated from one another by a Polytetrafluoroethylene (PTFE) spacer and the whole battery tank was sealed with a silicone rubber O-ring and a plastic crimp. Lithium foil was used as the anode and liquid lithium polysulfide solution was selected as catholyte using carbon felt as the current collector. The lithium foil, wrapped by a separator, was fixed on the negative bar with a snap joint structure to ensure good electrical contact. The separator here was used for electrical insulation of the carbon felt current collector from the lithium foil, which differs from the separator used in specially designed redox battery using expensive ion-selective membranes. The sealing requirement is not strict as the working temperature is low and no pressure is applied to the container. In contrast, the cost of LPS batteries here will be largely reduced, making it a good candidate for large-scale applications. For reactivation under stirring and heating condition, a magnetic stir bar was placed on the bottom of the tank. After configuring the carbon felt and lithium foil, lithium polysulfide (Li_2_S_8_) solution in 1,3-dioxolane (DOL)/1,2-dimethoxyethane (DME) was injected into the battery tank. Before sealing the battery, some additional sulfur powder was added to the bottom of the tank for future reactivation. As the polysulfide is in its highest order state, the additional sulfur on the bottom will not turn into soluble polysulfide, and is thus stored in the tank for future reactivation. The main idea of reactivation is to activate the dead sulfide species (Li_2_S_*x*_) on the lithium foil anode and carbon matrix cathode in order to recover its original capacity. When cycling at high mass loading of polysulfide caltholyte, dead sulfide species are easily deposited on the surface of lithium foil, as shown in Fig. 1c (*left*). The dark-red sulfide species is a mixture of Li_2_S_*x*_ compounds. These insulating species will block ion and electron transport and quickly deteriorate the capacity and stability of the LPS battery.

After reactivation, as shown in Fig. 1c (*right*), nearly all of the dead sulfide species were removed and the lithium foil surface is refreshed. The reactivation process not only increases the cell capacity, but also reduces the impedance of the battery thus enhancing the cell stability. Based on the outlined new cell configuration design, a high performance LPS battery was achieved.

LPS flow battery with reactivation function was designed based on a new battery configuration. Figure 1d (*left*) shows a schematic of the LPS flow battery system. The whole energy storage system can be divided into three parts: polysulfide storage tank, heating and stirring tank, and battery tank. The flow battery system can work in two modes: continuous flow working mode and intermittent flow working mode. In the continuous mode, the pump remains on and the polysulfide electrolyte continuously flows through the battery tank. The dead sulfide species on the lithium foil and solid precipitates on the carbon felt current collector can be re-dissolved or reactivated with the flowing polysulfide solution. The stirring and heating tank helps to reactivate the dead sulfide species and solid precipitates. As for the intermittent flow working mode, the pump alternates between the on and off state. After a certain period of cycling, the pump is turned on to initiate the reactivation process and inject fresh polysulfide solution. Intermittent flow working mode may be more suitable for practical application as the pump consumes fewer additional electricity.

To further demonstrate the LPS flow battery system, a real system was set up using the battery tank described above to simulate this mode. As shown in Fig. 1d (*right*), the whole system includes electrolyte storage tank, battery tank, circulation tube, magnetic circulation pump, hot plate with stirring function, and load (e.g., bike light). The electrolyte storage tank was placed on the hot plate and served as the polysulfide reactivation and storage vessel. The battery tank was assembled with carbon felt, lithium foil, and separator. Fresh and warm polysulfides can be injected into the battery tank through the pump, and the dead sulfide species can be reactivated in the storage tank under stirring and heating condition.

### Dead sulfide species reacting with sulfur powder

**Fig. 2:**
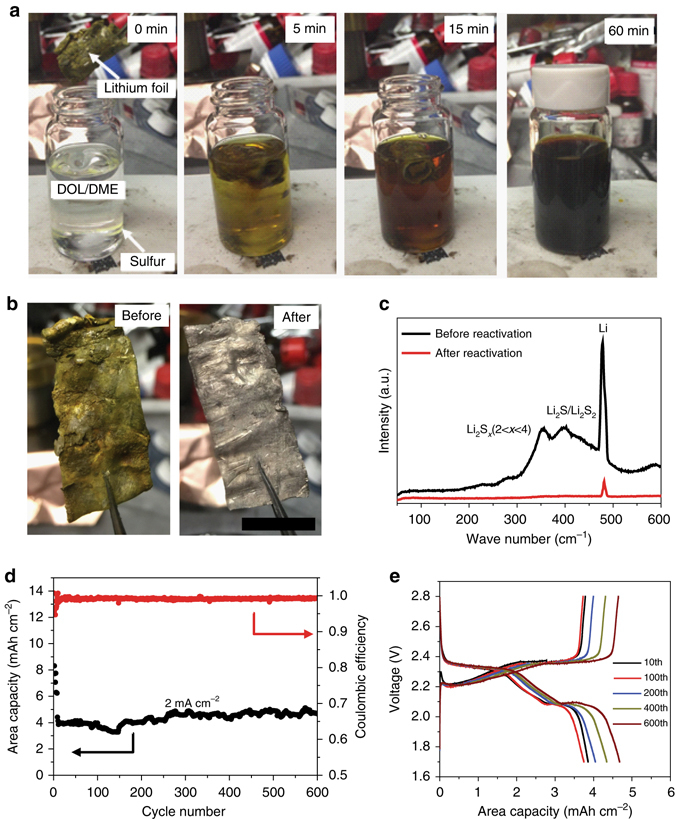
Dead sulfide species reaction with sulfur powder. **a** Optical images of dead sulfide species on the surface of lithium foil after reaction with sulfur powder in DOL/DME electrolyte under stirring and heating (70 °C) condition to form polysulfides after 0, 5, 10, and 60 min. **b** Optical images of lithium foil before and after dead sulfide species reaction with sulfur powder. (*Scale bar*, 2 cm) **c** Raman measurement of lithium foil before and after dead sulfide species reaction with sulfur powder. **d** Electrochemical performance of lithium foil (after reactivation) with 5 M LPS catholyte. **e** Voltage profiles of lithium foil (after reactivation) with 5 M LPS catholyte

To further confirm the reaction of dead sulfide species with sulfur powder under stirring and heating condition, sulfide species evolution experiments were conducted by stirring and heating lithium foil deposited with dead sulfide species in DOL/DME electrolyte with sulfur powder. As shown in Fig. 2a, a bottle of DOL/DME (1:1) electrolyte with a small amount of sulfur powder (300 mg) added at the bottom was placed on a hot plate heating at 70 °C in an argon-filled glove box (H_2_O < 0.1 ppm and O_2_ < 0.1 ppm). Then the lithium foil with dead sulfide species on the surface was immersed into the electrolyte. After stirring and heating, the dead sulfide species dissolved into the electrolyte and the color becomes darker and darker. About 1 h later, the solid dead sulfide species were nearly all dissolved and the electrolyte becomes dark red. Then the lithium foil was taken out and washed with DOL to remove liquid polysulfide on the surface. No obvious solid precipitates are observed in the electrolyte solution (Fig. 2a, 60 min). Figure 2b shows the optical image of lithium before and after reaction with sulfur powder. It is clear that nearly all the dead sulfide species were removed and the surface is refreshed and shining. The lithium foil remains intact and no obvious corrosion or deformation is noted. Further evidence is provided when lithium foil was immersed in high concentration polysulfide solution for a long time (the lithium foil used in this experiment was taken from a battery running for almost 4 months) and the morphology remains unchanged (Fig. 2b). Figure 2c shows the Raman measurement of lithium foil before and after reactivation. It demonstrates that there are Li_2_S_2_/Li_2_S and Li_2_S_x_ (2 < *x* < 4) species^32^ on the surface of the lithium foil before reactivation. After 1 h of heating and stirring, almost all of the species are reactivated and dissolved to form polysulfide solution. Only the lithium signal is detected after reactivation indicating the surface is very clean. To confirm the activity of lithium foil after reactivation, coin cells were assembled using reactivated lithium foil as anode and fresh lithium polysulfide electrolyte (5 M) as catholyte. At a current density of 2 mA cm^−2^, a high area capacity of 4 mAh cm^−2^ was measured even after 600 cycles (Fig. 2d). Voltage profiles show the standard discharge/charge process (Fig. 2e). Coin cells were also assembled using the newly dissolved lithium polysulfide electrolyte (from reaction of dead sulfide species with sulfur) as catholyte and fresh lithium foil as anode. The stable electrochemical performance (Supplementary Fig. 2) of the coin cell confirms the activity of the obtained lithium polysulfide solution. Stirring is very important during the reactivation process. In the procedure for preparing the polysulfide solution, both stirring and heating are necessary for the formation of a uniform solution. In our case, as the dead sulfide species are deposited on the surface of lithium foil, stirring can increase the reaction rate and effective contact area between dead sulfide species and sulfur powder. Based on our experiment, the process can be completed very quickly, requiring less than an hour.

Another experiment was conducted to verify the stirring and heating function. Three vials were prepared with Li_2_S and Sulfur powder (1:7, mass ratio, Supplementary Fig. 3). Vial 1 was neither heated nor stirred, vial 2 was only stirred while vial 3 was both heated (70 °C) and stirred. One hour later, almost all the sulfur powder in vial 3 reacted to form soluble Li_2_S_8_. In sharp contrast, vial 1 and 2 still contain a large quantity of solid powder at the bottom of the vial. Even after 48 h, the Li_2_S has not completely reacted with sulfur Stirring can accelerate the reaction but heating is essential in forming a uniform solution. This demonstrates that the heating and stirring process in the battery tank plays an important role in reactivation, which cannot be realized in coin cells or pouch cells. It is noted that this process engineering is compatible with large-scale energy storage station construction.

### Characterization of dead sulfide species

**Fig. 3:**
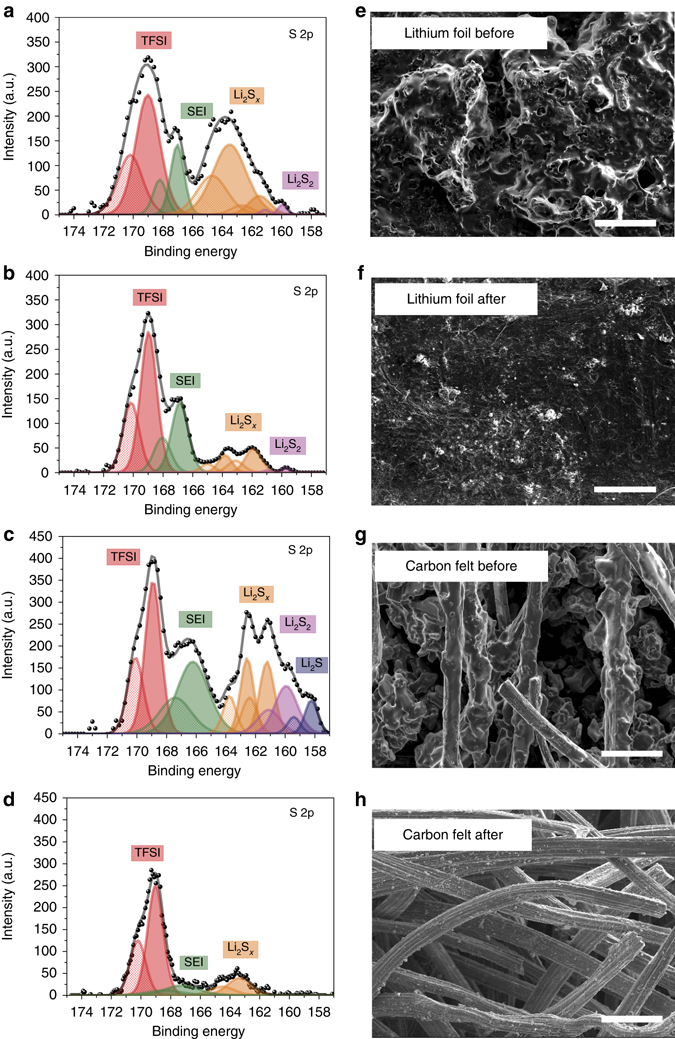
Characterization of dead sulfide species. **a**–**d** XPS analysis before and after reactivation: **a**, **b** lithium foil, **c**, **d** carbon felt. S_2p_ spectra are presented, including peak deconvolution and assignments. **e**–**h** SEM images before and after reactivation: **e**, **f** lithium foil (*scale bar*, 100 μm), **g**, **h** carbon felt (*scale bar*, 50 μm)

In order to understand the mechanisms for capacity improvement during the reactivation process, we carried out X-ray photoelectron spectroscopy (XPS) and scanning electron microscopy (SEM) measurements of lithium foil and carbon felt before and after reactivation. Before reactivation, the battery was cycled for 50 cycles and was stopped at the end of the discharge process (2.06 V). The high-resolution S 2p region spectra are shown in Fig. 3a–d, corresponding to the surface species on the lithium foil and carbon felt before and after reactivation. All the binding energies were calibrated with respect to the C1s peak at 284.6 eV. Spin–orbit coupling gives rise to a doublet in the S 2p peak ((2p_1/2_−2p_3/2_)) separated by 1.18 eV with a 2/1 intensity ratio^33^. Signal from Bis(trifluoromethane) sulfonimide lithium salt (LITFSI) in the electrolyte can be observed on both lithium and carbon felt (S 2p_3/2_ at 169.4 eV) due to the electrolyte precipitate trapped on the rough surfaces^34, 35^. Additional 2p_3/2_ peaks at 167 eV are assigned to the degradation product of Bis(trifluoromethane) sulfonimide (TFSI), which forms a solid–electrolyte interphase (SEI) on the electrode^28, 33^. The presence of soluble polysulfide (Li_2_S_x_) can be verified from peaks at ~162 and 164 eV, together with Li_2_S_2_/Li_2_S peaks at 160 and 158.3 eV^36^. The non-soluble species generated from the reduction of longer-chain polysulfide^37^ will cause a loss of reversible capacity in the following cycles of the polysulfide catholyte. After the reactivation process, the Li_2_S_2_/Li_2_S signal diminishes on both the lithium (Fig. 3a, b) and carbon electrodes (Fig. 3c, d), indicating the dead sulfide species have dissolved and reacted back into the catholyte. Additional differences between lithium foil and carbon felt before and after reactivation can be found in the C1s spectra (Supplementary Figs. 4 and 5). The polysulfide (Li_2_S_*x*_) signal was largely reduced after reactivation, which are mainly due to the SEI component changing. The SEM images of lithium foil and carbon felt before/after activation, in Fig. 3e–h validate this smoothing effect. In sum, the heating process can reactivate the sulfur species that precipitated on both anode and cathode and increase the accessible surface area on the cathode current collector, ultimately improving the cell capacity.

### Electrochemical performance

**Fig. 4:**
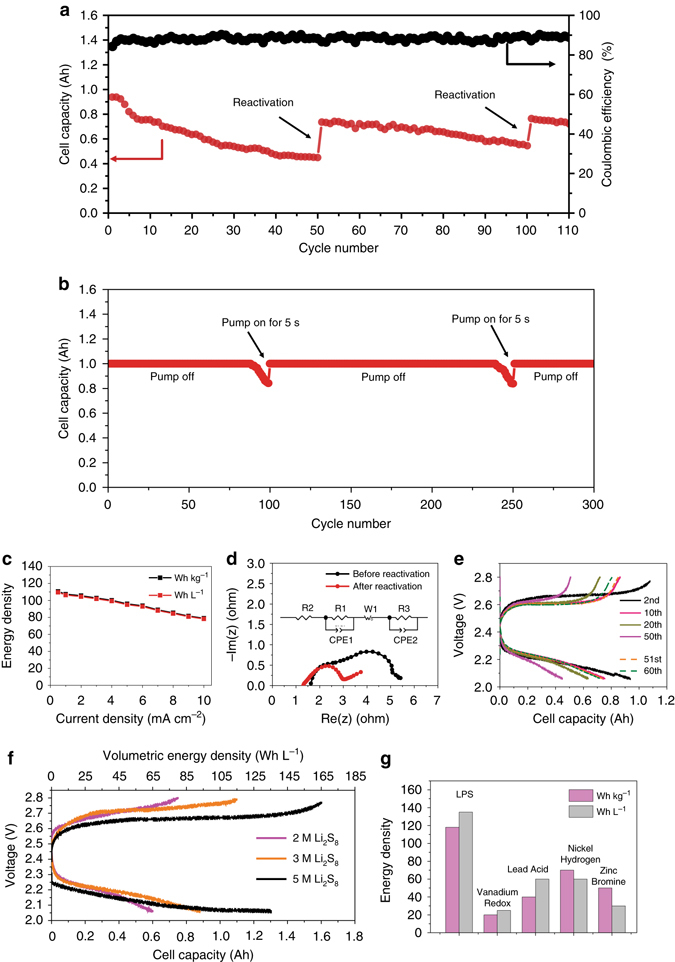
Electrochemical characterization of LPS battery. **a** Cell capacity and Coulombic efficiency of single LPS battery during 110 cycles (reactivation was conducted every 50 cycles). **b** Constant capacity cycling of LPS flow battery system (recipe of LPS flow battery system can be seen in Supplementary Tables 5 and 6). The charge capacity is set to 1000 mAh and the discharge cutoff voltage is set to 2.06 V. **c** Gravimetric and volumetric energy density at different current densities. **d** EIS measurement of LPS battery before and after reactivation (Inset Equivalent circuit diagram of LPS battery). **e** Voltage profiles and the corresponding cell capacity before and after reactivation. **f** Voltage profiles and their corresponding cell capacity and volumetric energy density for different concentrations of Li_2_S_8_. (2 and 3 M with pure carbon felt and 5 M with activated carbon felt, which has higher surface area for active materials trapping, as the current collector). **g** Gravimetric and volumetric energy density comparison between different batteries designed and used for grid application

After effective reactivation, our LPS battery shows excellent electrochemical performance. As demonstrated in Fig. 4a, at a high current density of 5 mA cm^−2^, a high single-cell capacity of 0.9 Ah was achieved (recipe can be seen in Supplementary Table 2, additional 0.4 g sulfur powder was added for reactivation). Previous reports of LPS battery using coin cells demonstrate a capacity of just 1–3 mAh, which is two orders lower than our results. Even though the cell capacity decays due to the formation of dead sulfide species, leading to irreversible capacity, the capacity can be immediately recovered to a higher value after reactivation that was conducted every 50 cycles under stirring and heating at 70 °C. The increased capacity comes from the newly formed soluble polysulfide and it can recover as much as 50% of the lost capacity. Even after 110 cycles, the battery tank still retains a high capacity of 0.7 Ah, which clearly confirms that reactivation can improve the performance of LPS batteries. Not only can the dead sulfide species on the metallic lithium anode be reactivated, but also those on the carbon felt cathode. Even though the voltage window was controlled between 2.06 and 2.8 V, some Li_2_S/Li_2_S_2_ species are inevitably formed as the disproportionation reaction occurs. Reactivation of these species increases the overall cell capacity, while simultaneously enhancing the conductivity of the carbon felt. We further test the LPS flow system with reactivation equipment as shown in Fig. 1d. The LPS flow battery system can be charged and discharged normally at a current of 200 mA in the constant capacity cycling mode (Supplementary Fig. 6 and Supplementary Movie 1). Reactivation induced by turning the pump on (intermittent flowing mode) was conducted when the capacity decayed, as demonstrated in Fig. 4b. After reactivation, the flow battery system can run for another hundred cycles. Fresh and warm polysulfide was injected into battery tank from the reactivation tank, which helps react with the dead sulfide species and increase the capacity. On the other hand, old polysulfide was circulated into the reactivation tank for further reactivation, thus turning into fresh polysulfide with high activity. Recipe of LPS flow system can be seen in Supplementary Tables 3 and 4.

## Discussion

The new setup, which is similar to the industrial standard, can significantly increase both the energy density and the mass loading. Figure 4c shows the energy density at different current densities. Even at a high current density of 9 mA cm^−2^, the specific and volumetric energy can still reach 82 Wh kg^−1^ and 80 Wh L^−1^, much higher than our previous results (72 Wh L^−1^) measured in a coin cell configuration with relatively low mass loading and low current density (1.5 mA cm^−2^). Since dead sulfide species lower the electronic and ionic conductivity, the reactivation process decreases the charge transfer resistance, which is supported by the Nyquist plot obtained from electrochemical impedance spectroscopy (EIS). Figure 4d shows EIS result of LPS battery before and after reactivation (lithium foil anode, 3 M Li_2_S_8_ as initial catholyte, and carbon felt as cathode current collector). The equivalent circuit diagram of LPS battery, which was fitted well with the experimental result, was shown in the inset of Fig. 4d. Before reactivation, the value of R1 that represents the interfacial impedance of lithium was 1.321 Ω, which was reduced to 0.52 Ω after reactivation. The surface kinetics of activated lithium foil is much faster than the lithium foil pre-activation. The EIS measurement provides strong evidence for the improvement of electrochemical stability through reactivation (Fig. 4d, Supplementary Fig. 7). Figure 4e shows the voltage profiles with single-cell capacity before and after reactivation. In the 51st cycle, the cell capacity increases by about 50% compared with that of the 50th cycle. The discharge profile in the 60th cycle is almost the same as in the 50th cycle, meaning there is almost no capacity decay during the following cycles. It is noted that the single-cell reactivation process can be completed in about 2 h (Supplementary Fig. 8). Figure 4f shows the voltage profiles with single-cell capacity and volumetric energy density at different concentrations of Li_2_S_8_. As expected, with increasing concentration, cell capacity and volumetric energy also increase. At 5 M concentration, a high energy density of 135 Wh L^−1^ was attained when employing activated carbon felt (using NaOH as an activation agent) as a current collector with high surface area. The mass loading of each concentration is very high. About 1–2.6 g sulfur element (sulfur and sulfur in Li_2_S) was used per single battery (Supplementary Tables 5 and 6). Compared with other battery technologies designed or used for grid scale energy storage^38, 39^, LPS batteries demonstrate obvious advantages in energy density. The volumetric energy density is four times higher than that of vanadium redox flow battery, and two times higher than lead acid battery (Fig. 4g), making it a promising candidate for future large-scale energy storage applications.

A long cycle life, high capacity, and high energy density LPS battery was achieved by introducing a method for reactivation of dead sulfide species. It is noted that the battery tank design makes it very easy to assemble and disassemble. The replacement of lithium or adding sulfur is also operable in engineering practice (Supplementary Notes 3 and 4). The very low price of sulfur as a raw material makes it cost-effective for future large-scale storage for renewable energies like wind and solar. The energy cost was reduced to a low value (< 100$ kW h^−1^) (calculated based on Supplementary Table 7 and Supplementary Note 5, not including heating cost for reactivation). Through cost analysis, it can be seen that the cost of LITFSI contributes about half of the total cost (Supplementary Fig. 9). Low concentration LITFSI or cheaper alternative salts may reduce the energy cost to a much lower value (< 50$ kW h^−1^). This work successfully demonstrates the possibility of using our LPS battery for practical engineering application in grid energy storage.

## Methods

### Assembly of single LPS battery

An amount of 1.4 g sulfur powder and 0.28 g lithium sulfide were mixed in 16 ml DOL/DME (1:1) electrolyte in a 20 ml vial, followed by addition of 0.5 g LiNO_3_ and 1.5 g LITFSI. The LiNO_3_ and LITFSI were heated at 110 °C for 2 days before use. Then the solution was heated and stirred at 70 °C for 6 h to generate 3 M Li_2_S_8_ solution. Carbon felt was placed on the bottom of the negative part of the battery tank. Before use, the carbon felt was heated at 60 °C in a vacuum oven for 2 days. Then a magnetic stir bar and 0.4 g of sulfur powder were placed on the bottom of the tank. Lithium foil was fixed onto the negative bar with a snap joint structure to ensure good electrical contact and then wrapped with commercial separator (Celgard 2250). Lithium foil was set into the center of the carbon felt and Li_2_S_8_ solution was injected into the battery tank. Finally, the battery tank was sealed with a plastic crimp. There is a specially designed PTFE spacer between the positive part and negative part to insulate them from one another.

### Characterization

The electronic environment of the samples were investigated through XPS (Phi5000 VersaProbe, Ulvac-Phi) with Al Kα radiation. All samples were sealed in a vacuum transfer chamber in the glove box and then transferred into the XPS equipment for measurement. Raman was conducted through the Horiba Scientific LabRAM HR Evolution Spectrometer. The wavelength of laser is 532 nm and the power is 5 mW.

### Electrochemical measurement

The LPS battery was tested with a Biologic EC-Lab Electrochemistry instrument between 2.06 and 2.8 V. The applied current varies from 20 to 200 mA. EIS was conducted with frequency range of 100 kHz to 0.1 Hz, at the sinus amplitude of 5 mV.

### Assemble of LPS flow battery system

The demonstration LPS flow battery system was made with two battery tanks (one for the battery and one for polysulfide storage). The two tanks were connected through a 6 mm stainless steel tube. A magnetic circulation pump was connected to the two tanks. The battery tank assembly was the same as the above battery, and the electrolyte tank was injected with about 48 mL of polysulfide solution. The storage tank was placed on a hot plate for reactivation.

### Data availability

The data that support the findings of this study are available from the authors on reasonable request.

## Electronic supplementary material

Supplementary Movie 1

Supplementary Information
