# Supplementary material for: Reactivation of dead sulfide species in lithium polysulfide flow battery for grid scale energy storage
Source: Nat Commun. 2017 Sep 6;8:462. doi: 10.1038/s41467-017-00537-0 (PMC5587700; doi:10.1038/s41467-017-00537-0)
Supplement: Supplementary file 2 — Supplementary Information [file 41467_2017_537_MOESM2_ESM.pdf]

Description of Additional Supplementary Files

File Name: Supplementary Movie 1
Description: LPS semi flow system demonstration.

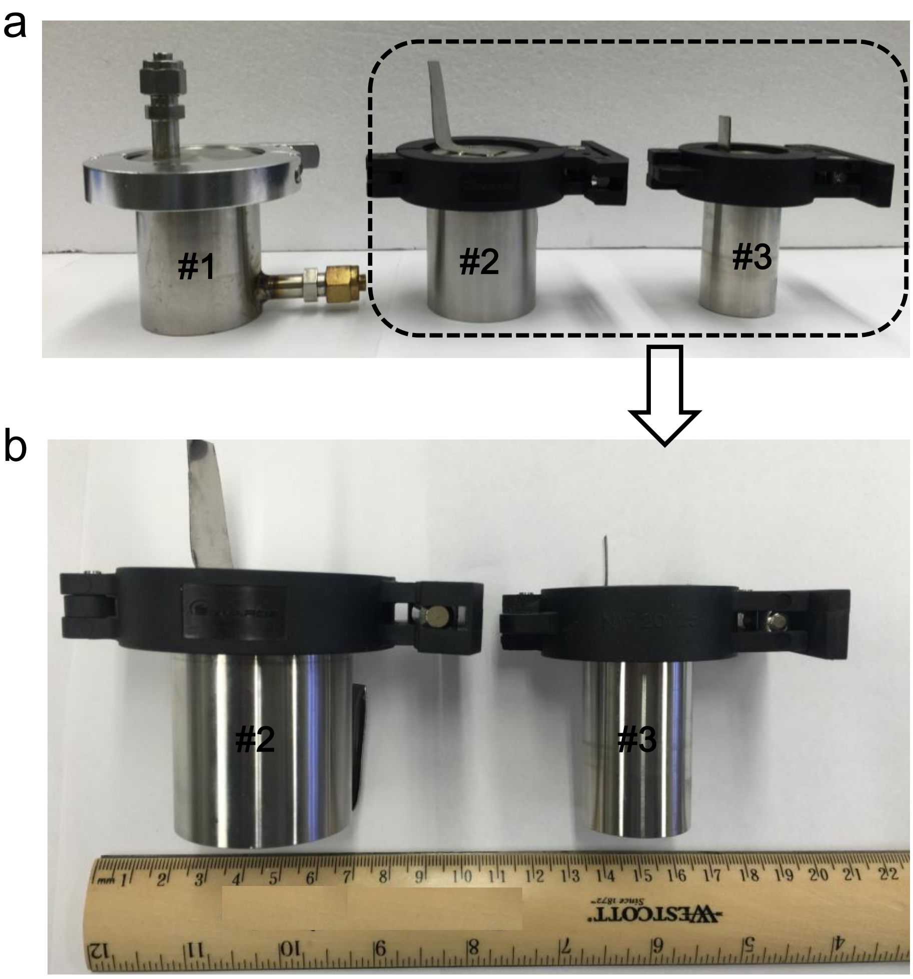

Supplementary Figure 1 | Image of different sized battery cases. (**a**) #1 for semi-liquid flow system, #2 and #3 for single cells. (**b**) #2 and #3 with a ruler as scaleplate.

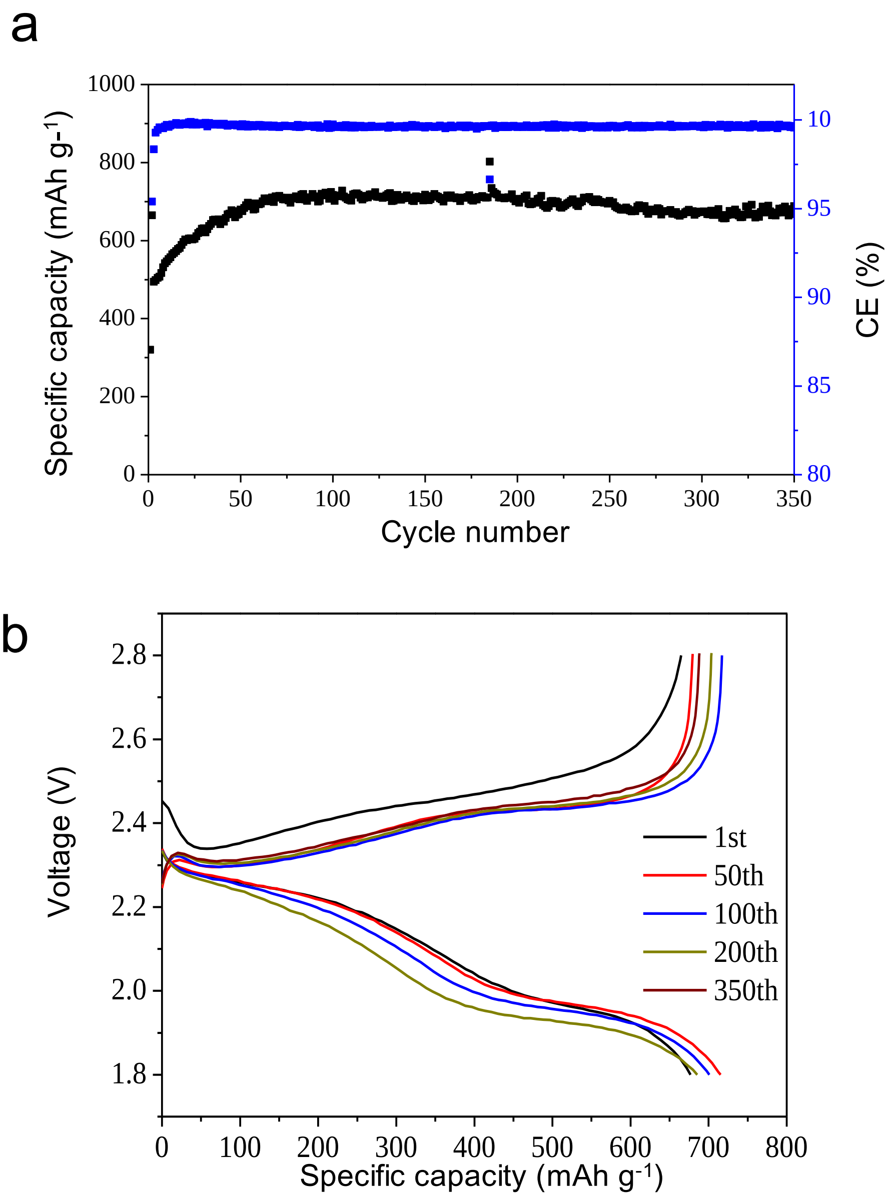

Supplementary Figure 2 | Electrochemical performance (coin cell) of newly dissolved lithium polysulphide electrolyte (from reaction of dead sulphide species with sulphur) as catholyte and fresh lithium foil as anode. (**a**) Cell capacity and coulombic efficiency during 350 cycles. (**b**) Voltage profiles of 1st, 50th, 100th, 200th, and 350th.

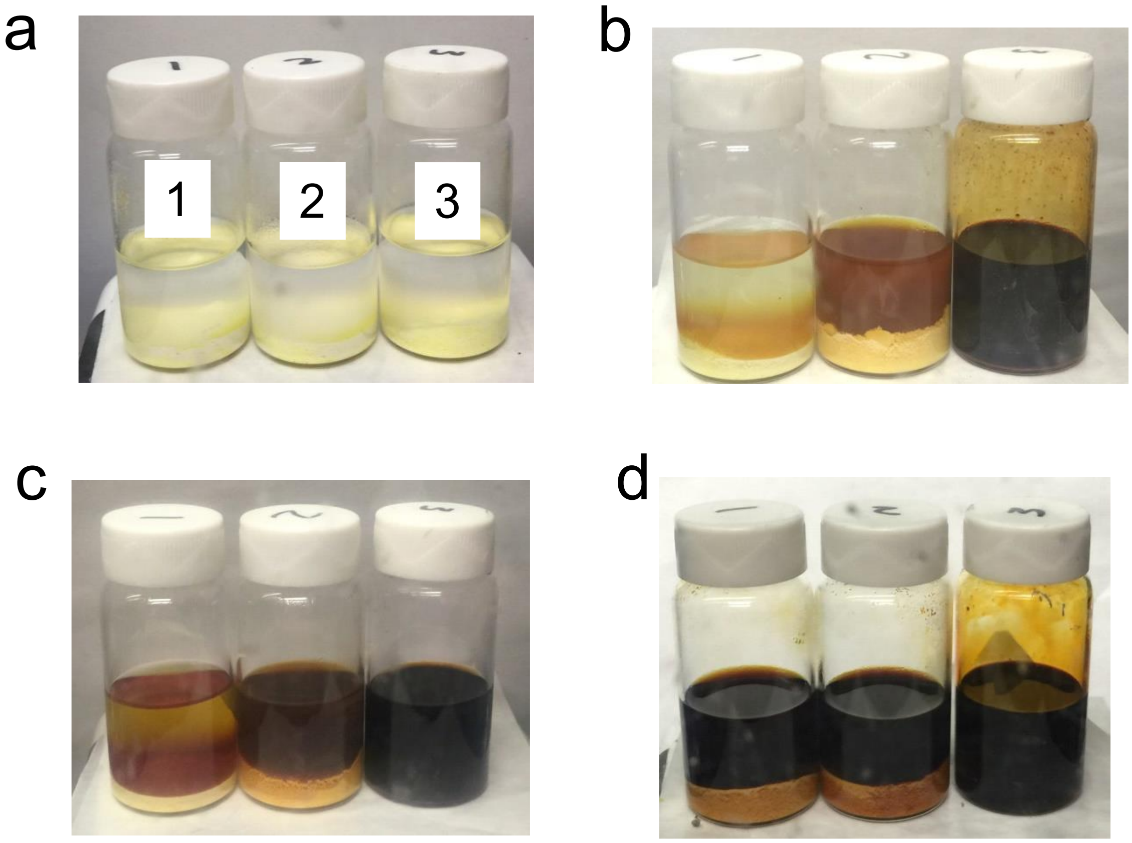

Supplementary Figure 3 | Images of polysulphide (Li_2S_8) solutions under different conditions. (**a**) Original sulphur powder with Li_2S powder mixed in DOL/DME solution (0 h). (**b**) The color change under different condition for 1 h. Vial 1 (without heating or stirring), Vial 2 (just stirring) and Vial 3 (with stirring and heating). (**c, d**) after 5 h and 48 h.

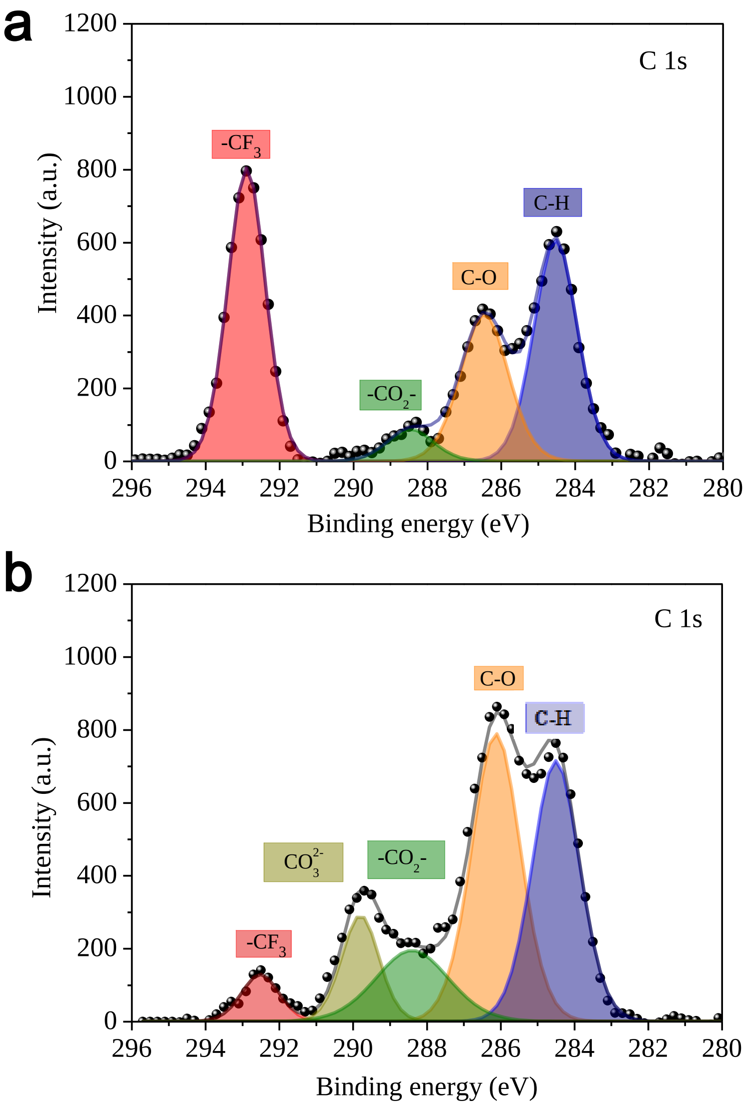

Supplementary Figure 4 | C1s spectra of lithium foil before (a) and after (b) reactivation.

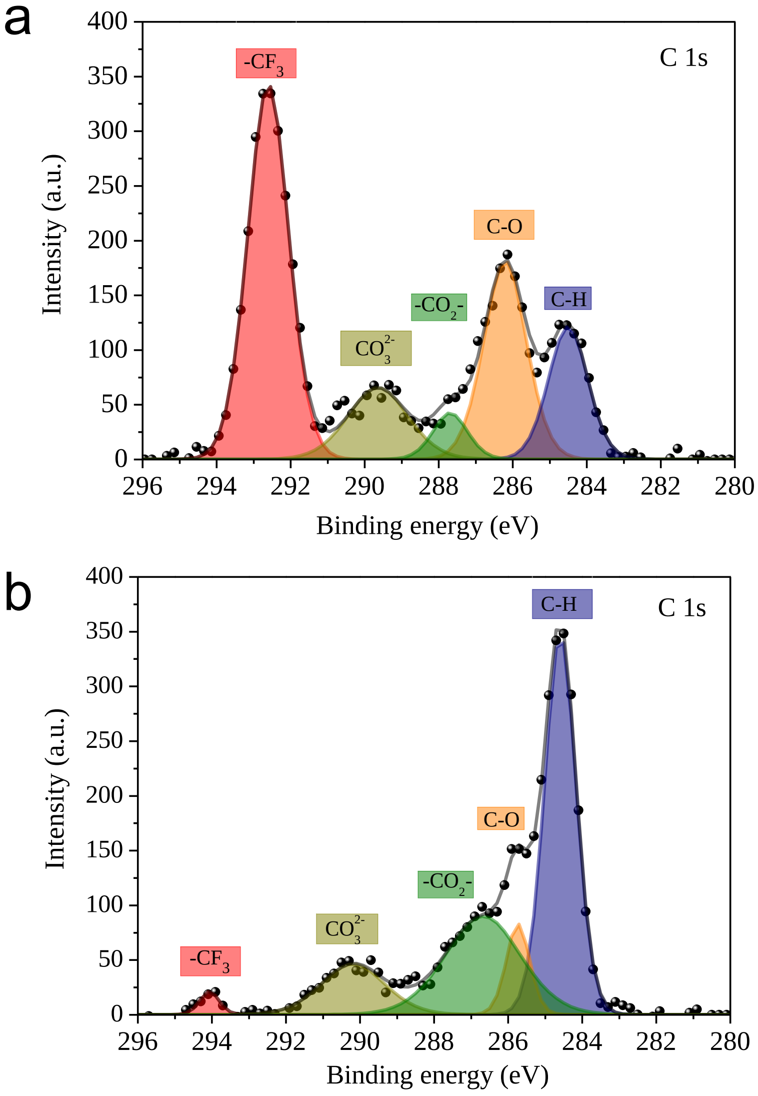

Supplementary Figure 5 | C1s spectra of carbon felt before (a) and after (b) reactivation.

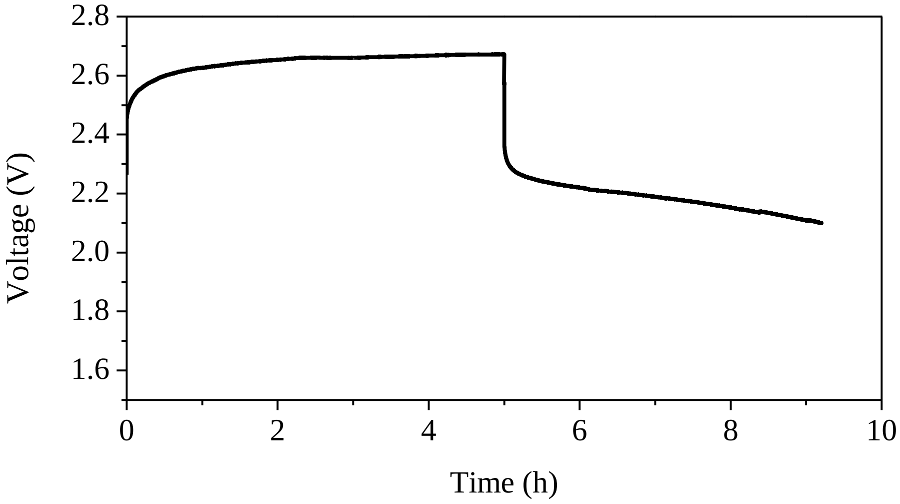

Supplementary Figure 6 | Charge-discharge voltage profile of LPS semi-liquid flow system.

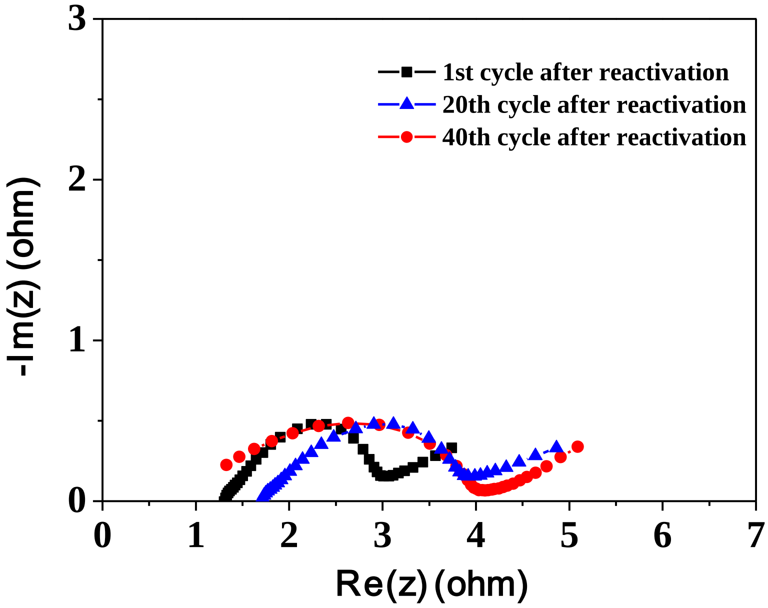

Supplementary Figure 7 | EIS measurement of LPS battery after reactivation.

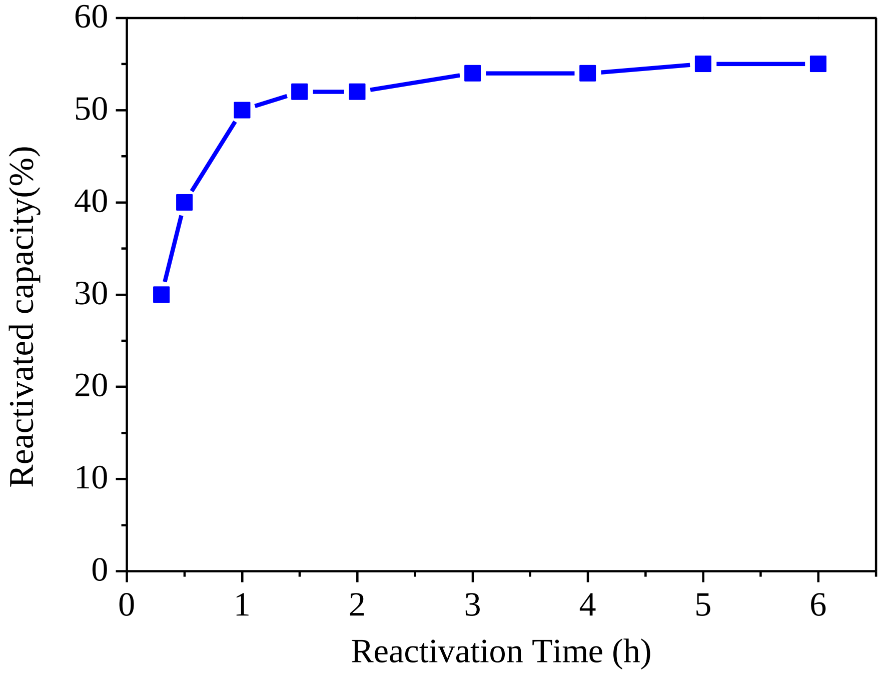

Supplementary Figure 8 | Reactivated capacity percentage at different reactivation times.

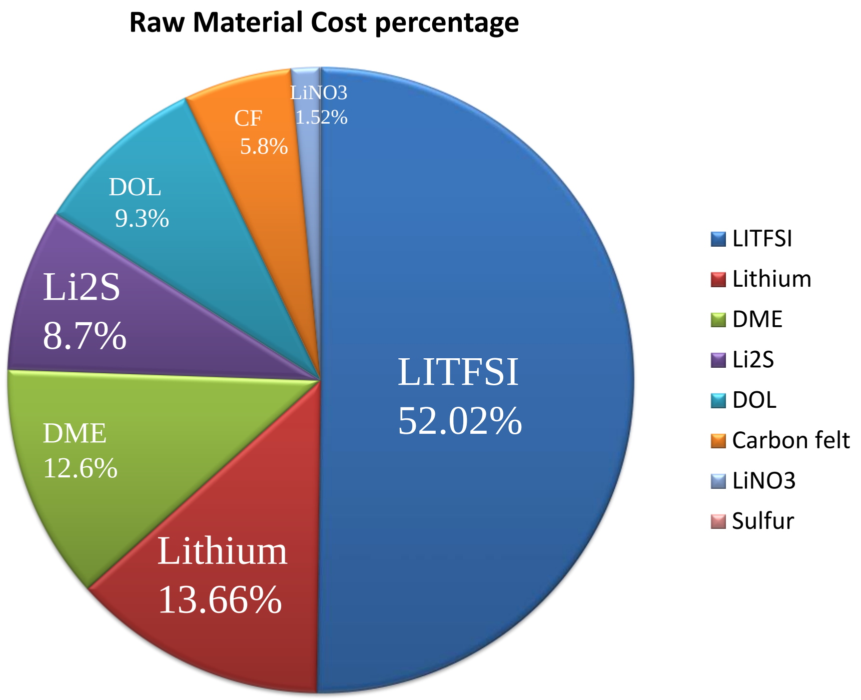

Supplementary Figure 9 | Cost percentage of raw materials used for LPS battery.

Supplementary Table 1 | Theoretical energy density of Lithium polysulfide battery

Li_2S_8	Wh L^{-1}	Wh kg^{-1}
2 M	61	60
3 M	91	86
4 M	120	112
5 M	149	135
6 M	178	157
7 M	206	178
8 M	234	198

Supplementary Table 2 | Recipe of battery cell (shown in Figure 4a and Figure 4f) using 3 M Li_2S_8.

Raw materials	Amount
S	1.4 g
Li_2S	0.28 g
DOL	8 mL
DME	8 mL
$LiNO_3$	0.5 g
LITFSI	1.5 g
Lithium foil	0.5 g
Carbon felt	1 g
Separator	0.1 g

Supplementary Table 3 | Recipe of LPS flow system (Battery tank, shown in Figure 1d) using 3 M Li_2S_8.

Raw materials	Amount
S	2.8 g
Li_2S	0.56 g
DOL	16 mL
DME	16 mL
$LiNO_3$	0.5 g
LITFSI	4 g
Lithium foil	0.8 g
Carbon felt	2 g
Separator	0.1 g

Supplementary Table 4 | Recipe of LPS flow system (Reactivation tank, shown in Figure 1d) using 3 M Li_2S_8.

Raw materials	Amount
S	4.2 g
Li_2S	0.84 g
DOL	24 mL
DME	24 mL
$LiNO_3$	0.75 g
LITFSI	6 g

Supplementary Table 5 | Recipe of battery cell (shown in Figure 4f) using 2 M Li_2S_8.

Raw materials	Amount
S	0.92 g
Li_2S	0.18 g
DOL	8 mL
DME	8 mL
$LiNO_3$	0.5 g
LITFSI	1.5 g
Lithium foil	0.5 g
Carbon felt	1 g
Separator	0.1 g

Supplementary Table 6 | Recipe of battery cell (shown in Figure 4f) using 5 M Li_2S_8.

Raw materials	Amount
S	2.3 g
Li_2S	0.46 g
DOL	8 mL
DME	8 mL
$LiNO_3$	0.5 g
LITFSI	2 g
Lithium foil	0.5 g
Carbon felt	1 g
Separator	0.1 g

Supplementary Table 7 | Raw materials' cost

Raw materials	Cost
S	150 \$ Ton^{-1}
Li_2S	80,000 \$ Ton^{-1}
DOL	3,000 \$ Ton^{-1}
DME	4,600 \$ Ton^{-1}
$LiNO_3$	7,700 \$ Ton^{-1}
LITFSI	82,000 \$ Ton^{-1}
Lithium foil	100,000 \$ Ton^{-1}
Carbon felt	15,000 \$ Ton^{-1}
Separator	1.5 \$ m^{-2}

Supplementary Note 1: The theoretical energy density calculation. The theoretical energy density can be calculated based on the following equations:

(1) Volumetric energy density (Wh L^{-1})

$$E(\text{volumetric}) = \frac{C_{PS,v} * C_{Li,v}}{C_{PS,v} + C_{Li,v}} * V_{average\ voltage} \quad (1)$$

(2) Gravimetric energy density (Wh kg^{-1})

$$E(\text{gravimetric}) = \frac{C_{PS,g} * C_{Li,g}}{C_{PS,g} + C_{Li,g}} * V_{average\ voltage} \quad (2)$$

$C_{PS,v}$ stands for the theoretical volumetric capacity of lithium polysulfide catholyte;

$C_{Li,v}$ stands for the theoretical volumetric capacity of lithium metal anode;

$C_{PS,g}$ stands for the theoretical gravimetric capacity of lithium polysulfide catholyte;

$C_{Li,g}$ stands for the theoretical gravimetric capacity of lithium metal anode;

$C_{PS,v} = qmc$, where q is the specific capacity for the polysulfide active material based on the mass of sulphur. For redox reactions between S and Li_2S_4, q=418 mAh g^{-1}. m is the molar weight of sulphur, 32 g mol^{-1}. C is the concentration of polysulfide in the unit of mole of sulphur per liter. For 5M lithium polysulfide electrolyte, $C_{PS,v} = 66.9\ \text{mAh}\ \text{cm}^{-3}$.

$C_{PS,g} = \frac{C_{PS,v}}{\rho}$, where ρ is the density of the catholyte. $\rho = (m_{Li_2S} + m_S + m_{LiNO_3} + m_{LITFSI} + m_{DOL} + m_{DME})/(V_{DOL} + V_{DME})$

$C_{Li,g} = 3861\ \text{mAh}\ \text{g}^{-1}$

$C_{Li,v} = C_{Li,g} * \rho = 3861 \frac{mAh}{g} * 0.534\ \text{g cm}^{-3}$=2061.7 mAh cm^{-3}

$V_{average\ voltage} = 2.30\ \text{V}$ based on the discharge voltage profile calculation

Supplementary Note 2: Real energy density calculation of LPS battery. The theoretical energy density was calculated based on the real capacity, real total volume and real total mass.

$$E(\text{Real Volumetric}) = \frac{\int_0^t I*\text{U}}{V_{Total\ Volume}} \tag{3}$$

$$E(\text{Real Gravimetric}) = \frac{\int_0^t I*\text{U}}{M_{Total\ Mass}} \tag{4}$$

t stands for the discharge time in one cycle.

I stands for the discharge current in one cycle.

U stands for the instant discharge voltage in one cycle.

$V_{Total\ Mass}$ stands for the total volume of our LPS battery.

$$V_{Total\ Mass}=V_{Carbon\ Felt}+V_{Catholyte}+V_{Lithium\ Foil} \tag{5}$$

$V_{Total\ Mass}$ stands for the total mass weight of our LPS battery.

$$M_{Total\ Mass}=M_{Carbon\ Felt}+M_{Catholyte}+M_{Lithium\ Foil}+M_{Separator} \tag{6}$$

Supplementary Note 3: Calculation of additional sulphur powder for reactivation. The recipe of LPS battery cell is provided in the supplementary information (Supplementary Table 2). In a 3 M Li_2S_8 cell configuration, the amount of lithium (including lithium foil and lithium in Li_2S) was 0.58 g and the amount of sulphur (including sulphur powder and sulphur in Li_2S) is 1.6 g. In our lithium-polysulphide battery system the desired discharge product is highly soluble Li_2S_4 for semi-flow batteries. Assuming that all the sulphur is converted into Li_2S_4, the total consumption of lithium is 0.175 g. Based on this data the excess lithium in the battery was 0.405 g. The excess lithium provides the possibility of precipitation of insoluble low order polysulphides. In our experiment, the remediation amount, which refers to the quantity of additional sulphur which was added to the battery was not determined by the excess amount of lithium, because not all the lithium will participate in the formation of low order polysulphide precipitation as the discharge voltage was fixed above the formation of Li_2S. Here the remediation amount was estimated by the real capacity loss. As can be seen from Figure 4a, after 50 cycles the capacity loss can reach approximately 0.5 Ah. An additional 0.4 g of sulphur powder (according to an additional capacity of 0.64 Ah) was added for reactivation. During the 110 cycles, reactivation was conducted every 50 cycles (twice in total). After reactivation, the capacity of the cell was increased by about 0.25 Ah. In the long term, when lithium is

continuously consumed, remediation can be estimated by the excess amount of lithium (0.405 g), which requires an additional 3.7 g of sulphur based on soluble Li_2S_4 formula. In a real battery situation, the cell capacity loss cannot be totally attributed to low order polysulphides precipitation. Here the reactivation process not only removes the polysulphides precipitates, but also improves the catholyte homogeneity, decreases cell impedance and enhances reaction kinetics, which helps recover the cell capacity. Therefore, the real remediation amount (additional sulphur) will be less than 3.7 g.

Supplementary Note 4: Lithium consumption. With remediation carried out extensively, the lithium will be continuously converted to polysulphides. Indeed, there is a possibility that all the lithium metal at the negative electrode is consumed. Based on our experiment, this consumption rate is very slow so that enough lithium metal is left even after cycling for a long time. The electrochemical test in Figure 4a lasts for 4 months with additional 0.4 g sulphur added for reactivation. In view of the chemical reaction, at least 0.043 g lithium metal was needed to convert 0.4 g of sulphur into soluble Li_2S_4. Based on the recipe of battery cell provided in the supplementary information (Supplementary Table 2), the excess lithium metal amount in the battery was 0.405 g. This means the excess amount of lithium can support this rate of lithium metal consumption for 38 months (almost 3 years). It is noted that here the lithium foil used is very thin, in practical employment if the lithium metal amount was doubled or even more, it is possible for the cycle life of LPS system to be doubled or tripled to nearly 10 years (the projected lifetime of a grid energy storage station). On the other hand, the replacement of lithium foil is possible as opening the LPS system is simple. In general, both employment of thick lithium foil and replacement of lithium foil can guarantee the service life of the lithium metal anode.

Supplementary Note 5: The energy cost calculation. The prices of raw materials are obtained from www.alibaba.com. Even though the polysulfide solution can be synthesized through direct reaction between sulphur and lithium which will be cheaper than the fairly expensive Li_2S, here we calculate the energy price based on all the raw materials that were used in our experiment. Supplementary Table 2 lists the raw materials' market cost. It is obvious that Li_2S, LITFSI and $LiNO_3$ are among top three most expensive raw materials. According to the amount used in a LPS battery, although $LiNO_3$ and Li_2S are expensive, they will not contribute to the total price significantly. To our surprise, LITFSI contributes more than 50% of the total price, which indicates that the use of 1 M concentration LITFSI is not cost-effective. Based on the capacity measured in our experiment and the raw materials cost, the calculated real energy cost is 98 \$ kWh^{-1}. This price will be a little bit lower if the polysulfide solution is synthesized through direct reaction between sulphur and lithium. It should also be noted that if we reduce the concentration of LITFSI (for example 0.5 M), the energy cost will be about 60-70 \$ kWh^{-1}.
